# Use of dual priming oligonucleotide system-based multiplex RT-PCR assay to detect five diarrhea viruses in pig herds in South China

**DOI:** 10.1186/s13568-021-01255-z

**Published:** 2021-07-01

**Authors:** Guangbin Si, Jiawei Niu, Xia Zhou, Yongsheng Xie, Zhifei Chen, Gen Li, Ruiai Chen, Dongsheng He

**Affiliations:** 1 Key Laboratory of Zoonosis Prevention and Control of Guangdong Province, College of Veterinary Medicine, South China Agriculture University, Guangzhou, China; 2 Key Laboratory of Biotechnology and Bioproducts Development for Animal Epidemic Prevention, Ministry of Agriculture, Zhaoqing, Guangdong China; 3 Guangdong Enterprise Key Laboratory of Biotechnology R&D of Veterinary Biologics, Zhaoqing, Guangdong China; 4 Zhaoqing Dahuanong Biology Medicine Co., Ltd, Zhaoqing, Guangdong China

**Keywords:** Dual priming oligonucleotide (DPO), Multiplex RT-PCR method, Swine enteric viruses, Diagnosis

## Abstract

In this study, a specific and simple method based on the dual priming oligonucleotide (DPO) system was developed to simultaneously detect transmissible gastroenteritis virus (TGEV), porcine epidemic diarrhea virus (PEDV), porcine rotavirus A (PRV-A), porcine delta coronavirus (PDCoV), and swine acute diarrhea syndrome coronavirus (SADS-CoV), associated with the major enteric RNA viruses in pigs. The DPO system-based multiplex RT-PCR method simplified the primer design and did not require optimization of the annealing temperature. Specificity analysis revealed that the method could specifically detect TGEV, PEDV, PRV-A, PDCoV, and SADS-CoV without any cross-amplification of other circulating swine viruses. The limit of detection of the method was as low as 10^3^–10^4^ copies/μL plasmid of each virus. The method also had good repeatability, and obvious results were seen in three repeat experiments with an interval of 45 days. This optimized multiplex RT-PCR method was used to evaluate 181 clinical swine samples that were collected from four provinces of China between September 2016 and August 2018. The results showed that the positive detection rates of PEDV, PDCoV, SADS-CoV, PRV-A, and TGEV were 30.94% (56/181), 17.67% (32/181), 11.6% (21/181), 9.39% (17/181), and 0.55% (1/181), respectively. Mixed infection of two or more viruses was also common. The DPO system-based multiplex RT-PCR could be a useful tool for detecting enteric virus infections. This method has the advantages of easy operation, low cost, high detection efficiency, and short running time for early diagnosis in clinical cases.

**Supplementary Information:**

The online version contains supplementary material available at 10.1186/s13568-021-01255-z.

## Key points

1. Swine enteric viruses that cause diarrhea caused enormous losses.
2. The DPO system-based multiplex RT-PCR method is critical for diagnosis.
3. Mixed infection of enteric viruses is common in the pig industry.

## Introduction

Diarrhea, especially viral diarrhea, seriously endangers the pig industry throughout the world; it is characterized by acute diarrhea, dehydration, and high mortality in the early stage of suckling piglets, and it results in severe economic losses in the global pig industry (Zhang et al. 2016). Transmissible gastroenteritis virus (TGEV), porcine epidemic diarrhea virus (PEDV), and porcine rotavirus A (PRV-A) are currently the most significant pathogens that cause diarrhea in piglets (Ding et al. 2020). In recent years, some coronaviruses have emerged or reemerged in the pig industry. Variant strains of PEDV were discovered in China in 2010, with a 100% death rate for piglets younger than 1 week old (Li et al. 2011). Porcine deltacoronavirus (PDCoV) was first detected in pig feces in Hong Kong in 2012 (Woo et al. 2012); subsequently, this virus spread, causing severe diarrhea and/or vomiting and atrophy in pigs in the United States (Li et al. 2014). In 2017, a novel swine enteric alphacoronavirus (SeACoV)—causing acute vomiting, watery diarrhea, and high mortality of newborn-piglets—was firstly reported in China (Gong et al. 2017; Pan et al. 2017). It was also named the porcine enteric alphacoronavirus (PEAV) (Xu et al. 2019) or the swine acute diarrhea syndrome coronavirus (SADS-CoV) (Zhou et al. 2018). For the sake of consistency, the term SADS-CoV is used to refer to this newly-emerging virus in current research (Yang et al. 2019).

These five swine enteric viruses cause similar clinical symptoms, and it is common for swine to be infected with two or more pathogens at the same time (Zhou et al. 2019), which makes clinical diagnosis difficult. Some detection methods, such as virus isolation and indirect immunofluorescence assay, are applied to detect viruses, but those methods are time-consuming and costly (Ding et al. 2020). Although polymerase chain reaction (PCR) is widely used in virus detection, as it is suitable for detecting large-scale samples, its disadvantages include difficulty in optimizing reaction conditions and weak specificity in the multiplex PCR reaction (Zhang et al. 2019; Zhou et al. 2019).

To solve these problems, a novel technology—dual priming oligonucleotide (DPO)—was developed. The DPO contains two separate priming regions joined by a polydeoxyinosine linker that assumes a bubble-like structure and is not involved in priming (Chun et al. 2007). The polydeoxyinosine linker confers the distinct annealing properties of the two priming regions. This structure makes the annealing temperature of primers insensitive, eliminating the need to optimize the annealing temperature of primers (Chun et al. 2007). The two priming regions are a longer 5′-segment that initiates stable priming and a shorter 3′-segment that determines target-specific extension (Chun et al. 2007). The advantage of DPO-based PCR reaction is the effective prevention of the amplification of non-specific templates. DPO technology has been applied to multiplex PCR and SNP-genotyping PCR of viruses or bacteria and has demonstrated the advantages of high sensitivity and specificity (Fan et al. 2017; Kim et al. 2008; Ma et al. 2015). Of the available PCR methods based on a DPO system to detect swine enteric viruses, none of them can differentiate these five viruses in one test.

In this study, we developed a DPO system-based multiplex RT-PCR assay for the differential detection of TGEV, PEDV, PRV-A, PDCoV, and SADS-CoV in the same reaction vial. This method simplified the primer design and had high sensitivity and specificity. Additionally, 181 swine intestinal samples taken in 2016–2018 in South China were analyzed. The results provided a detailed condition of mixed diarrhea infections; this will help formulate effective strategies to prevent diarrhea in piglets.

## Material and methods

### Viruses and clinical specimens

TGEV, PEDV, PDCoV, and SADS-CoV were previously isolated and identified by our laboratory and preserved at − 80 °C (GenBank accession number: DQ811788, AF353511, KY363867, MG605090). The VP7 of PRV-A (pig/China/NMTL/2008 G9P[23] strain, Accession number: JF781163) was synthesized by Beijing Qingke Biotechnology. A total of 181 swine intestinal samples were collected from commercial pigs with symptoms of diarrhea in the Guangdong, Guangxi, Jiangxi, and Fujian provinces in China between April 2016 and November 2018.

### Primer design

The whole genome of five enteric viruses retrieved from GenBank were aligned using Megalign software (DNAStar V7.0, Madison, WI, USA). Based on the well-conserved region of the whole genome sequence, a set of five specific DPO primers (Table 1) targeting the N gene of TGEV, PEDV, PDCoV, and SADS-CoV, as well as the VP7 gene of PRV-A, was designed. The information concerning the primers is shown in Table 1. The primers were calibrated to the final concentration of 10 μM and stored at − 20 °C.

**Table 1:** Details of the conventional primers and the DPO primers used in this study

Virus	Gene	Type	Sequence (5′→3′)	Amplicon
TGEV	N	C^a^	F^c^: GGTGGTTCTTCTACTACTTAGGTACTGGACCTCATG	472
			R^d^: ACATCACCTTTACCTGCAGTTCTCTTCCAGGTGTG	
		DPO^b^	F: GGTGGTTCTTCTACTACTTAGGIIIII^e^GACCTCATG	
			R: ACATCACCTTTACCTGCAGTTIIIIICCAGGTGTG	
PEDV	N	C	F: GGTGAGCGAATTGAACAACCTTCCAATTGGCATTTC	361
			R: GACCCTGGTTATTTCCACGATTCTGTGAATTACCAC	
		DPO	F: GGTGAGCGAATTGAACAACCTTCIIIIIGGCATTTC	
			R: GACCCTGGTTATTTCCACGATTCTIIIIITTACCAC	
PRV-A	VP7	C	F: CTCCTTTTAATGTATGGTATTGAATATACCACAG	123
			R: AAGAAATCTATAAATTATAAAGTCCATCGCACTAG	
		DPO	F: CTCCTTTTAATGTATGGTATTIIIIITACCACAG	
			R: AAGAAATCTATAAATTATAAAGIIIIICGCACTAG	
PDCoV	N	C	F: TCAACGCTAGAGGAAGACCTCAGGAGCGTGGAAGTG	263
			R: CATGATGCGAGGATCAGCCATACCCGTCTTCTCAG	
		DPO	F: TCAACGCTAGAGGAAGACCTCAGIIIIITGGAAGTG	
			R: CATGATGCGAGGATCAGCCATAIIIIICTTCTCAG	
SADS-CoV	N	C	F: GACCTGACTGTTGTTGAGGTTACTTCTAGAAGTGC	586
			R: TCCTAATTTGACTGGGTTTAGAGTAAGCCGAGACT	
		DPO	F: GACCTGACTGTTGTTGAGGTTAIIIIIAGAAGTGC	
			R: TCCTAATTTGACTGGGTTTAGAIIIIICCGAGACT	

^a^C means conventional primer

^b^DPO means dual priming oligonucleotide

^c^F: forward primers

^d^R: reverse primers

^e^I means deoxyinosine

### RNA extraction, reverse transcription, and construction of recombinant plasmids for standard

Viral RNA samples were extracted using Trizol reagent (Axygen, China), according to the manufacturer’s instructions. RNA was eluted into RNAse-free ddH_2_O and stored at − 80 °C until use. In this study, reverse transcription reactions of extracted RNA were performed with a PrimeScript™ 1st Strand cDNA Synthesis Kit (TaKaRa, Dalian, China).

The target fragment was obtained by amplification of cDNA with conventional primer and cloned into a pMD19-T vector (Takara, Dalian, China). The recombinant vector was transformed into DH5α *Escherichia coli* cells and shaken overnight at 37 °C; the cells were then purified with plasmid Mini Kit2 (Omega BioTek, USA), according to the manufacturer’s recommendations (Takara, Dalian, China). The positive recombinant plasmids were confirmed through sequencing analysis (Takara, Dalian, China). The recombinant plasmid concentrations were measured with UV spectrophotometry (NanoDrop one) and converted into copy numbers. The plasmid DNA was stored at − 80 °C until further use.

### The reaction of the DPO system-based multiplex RT-PCR

Before the DPO system-based multiplex RT-PCR was established, DPO system-based single RT-PCR assay for five viruses was established. The 50 μL consisted of the following: 25 μL of 2 × Accurate Taq Master Mix, 22 μL RNase free water, and 1 μL of DPO system set (10 μM) for each 1 μL of plasmid DNA template. The PCR condition was as follows: 94 °C for 30 s, followed by 35 cycles of 10 s at 98 °C, 30 s at 55 °C, 40 s at 72 °C, and with a final extension at 72 °C for 2 min. Amplified products were analyzed with 1.5% agarose gel electrophoresis.

For the DPO system-based multiplex RT-PCR assay, a single PCR assay was first established with the primer sets for TGEV. Then, different concentrations of PEDV, PDCoV, and SADS-CoV primer sets were added successively to the multiplex reaction system. The optimized primer concentration was directly applied to the reaction optimization of the subsequent virus. Finally, the PRV-A primer pair was added to the final multiplex PCR assay to optimize the primer concentration. In order to obtain better results for the multiplex PCR, the primer concentration and annealing temperature were optimized. PCR products were visualized on a 1.5% agarose gel.

### Comparison of annealing temperatures between DPO-primers and conventional primers

In order to test the effect of annealing temperature on the DPO primers and conventional primers, different annealing temperatures were used to evaluate the two kinds of primers. The annealing temperatures were 40 °C, 44 °C, 48 °C, 52 °C, 56 °C, and 60 °C, respectively.

### Specificity of the DPO system-based multiplex RT-PCR

The specificity of the DPO system-based multiplex RT-PCR was assessed in three steps. First, a single pair of the DPO primer was used to amplify the mixture of the five plasmid templates. Second, the mixture of the five DPO primers was used to amplify the templates of the five plasmid templates. Third, the specificity of the DPO system-based multiplex RT-PCR assay was evaluated through testing of the amplified products of porcine reproductive and respiratory syndrome virus (PRRSV), atypical porcine pestivirus (APPV), Seneca Valley virus (SVV), porcine circovirus 3 (PCV3), porcine respiratory coronavirus (PRCV), porcine teschovirus (PTV), and porcine parvovirus (PPV). The nuclease-free water served as a negative template.

### Sensitivity of the DPO system-based multiplex RT-PCR

To analyze the sensitivity of the established DPO system-based multiplex RT-PCR, the standard plasmids of TGEV, PEDV, PDCoV, SADS-CoV, and PRV-A that were prepared above were diluted in RNAse-free ddH_2_O. The initial concentrations of the five standard plasmids were 8.88 × 10^8^ copies/μL, 6.84 × 10^8^ copies/μL, 7.47 × 10^8^ copies/μL, 6.12 × 10^8^ copies/μL, and 6.51 × 10^8^ copies/μL, respectively. The plasmid mixtures from the five viruses were used as templates for PCR amplification within the DPO system-based multiplex reaction system. In addition, the sensitivity of each RT-PCR of the DPO system-based assay for the five viruses was evaluated with diluted plasmids. The plasmid of each virus was added to a single RT-PCR reaction of the DPO system as a template for amplification.

### Reproducibility of the DPO system-based multiplex RT-PCR

To evaluate the stability of the DPO system-based multiplex RT-PCR method and to eliminate the random error of single experimental results, multiple experiments were carried out on the basis of the optimized reaction conditions. Three repeated experiments were carried out with an interval of 45 days. Each time, three batches of plasmids were selected and mixed for DPO system-based multiplex RT-PCR reactions.

### Detection in clinical samples

A total of 181 swine intestinal samples were collected from the Guangdong, Guangxi, Jiangxi, and Fujian provinces in China between September 2016 and August 2018. All samples were diluted threefold with phosphate-buffered saline (PBS) using a vortex machine and were centrifuged at 4000×*g* 4 °C for 15 min. The total RNA of clinical samples was extracted by the above method. The supernatant was collected and used for RNA extraction and prepared as cDNA using a reverse transcription. Then, all cDNA were measured with the DPO system-based multiplex RT-PCR assay in this study.

## Results

### Establishment of the single RT-PCR of the DPO system-based assay

**Fig. 1:**
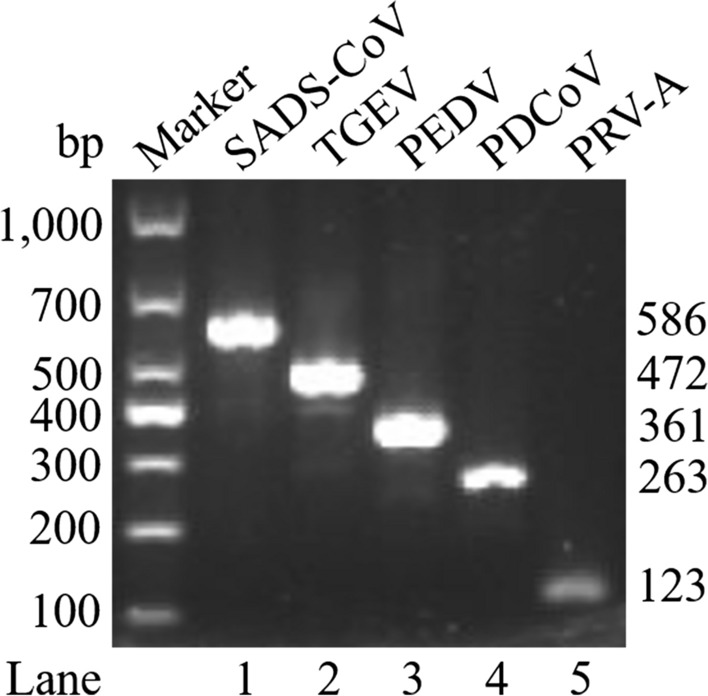
Development of a single DPO RT-PCR assay for detection of SADS-CoV, TGEV, PEDV, PDCoV, and PRV-A. M, Marker; Lane 1, SADS-CoV; Lane 2, TGEV; Lane 3, PEDV; Lane 4, PDCoV; Lane 5, PRV-A

The specific amplifications of SADS-CoV, TGEV, PEDV, PDCoV, and PRV-A were observed at the expected sizes of 586, 472, 361, 263, and 123 bp (Fig. 1), respectively. No other miscellaneous bands were produced, indicating the reliability and the specificity of the DPO primer sets. The accuracy of the amplified product was further confirmed with sequencing analysis.

### Establishment of the DPO system-based multiplex RT-PCR

**Fig. 2:**
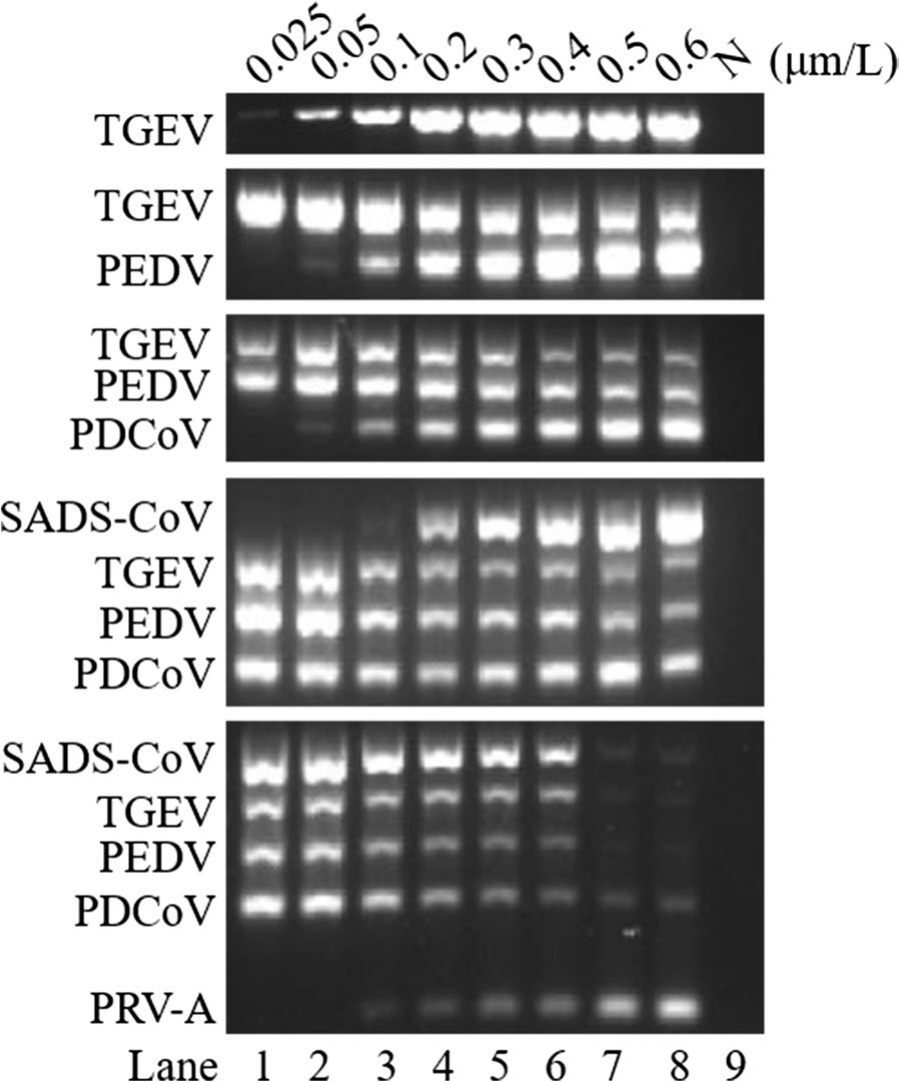
Optimization of the DPO system-based multiplex RT-PCR primer concentration. Lanes 1–8 represent the 8 primer concentrations 0.025 μM, 0.05 μM, 0.1 μM, 0.2 μM, 0.3 μM, 0.4 μM, 0.5 μM, and 0.6 μM; lane 9 represents negative control (N)

First, the optimal concentration of DPO primers for TGEV was determined (Fig. 2). Then, under the optimal working concentration of TGEV DPO primers, different concentrations of PEDV DPO primers were added in order to determine the best working concentration of PEDV DPO primers (Fig. 2). Thereafter, the DPO primer concentrations of PDCoV, SADS-CoV, and PRV-A were successively optimized according to the above methods. Finally, the optimal working concentration of DPO primers for the five viruses in the multiplex RT-PCR was determined (Fig. 2). The best final concentration of mixed DPO primers was as follows: 0.2 μM for TGEV, 0.3 μM for PEDV, 0.2 μM for PDCoV, 0.4 μM for SADS-CoV, and 0.4 μM for PRV-A.

### Comparison of annealing temperatures between DPO-based primers and conventional primers

**Fig. 3:**
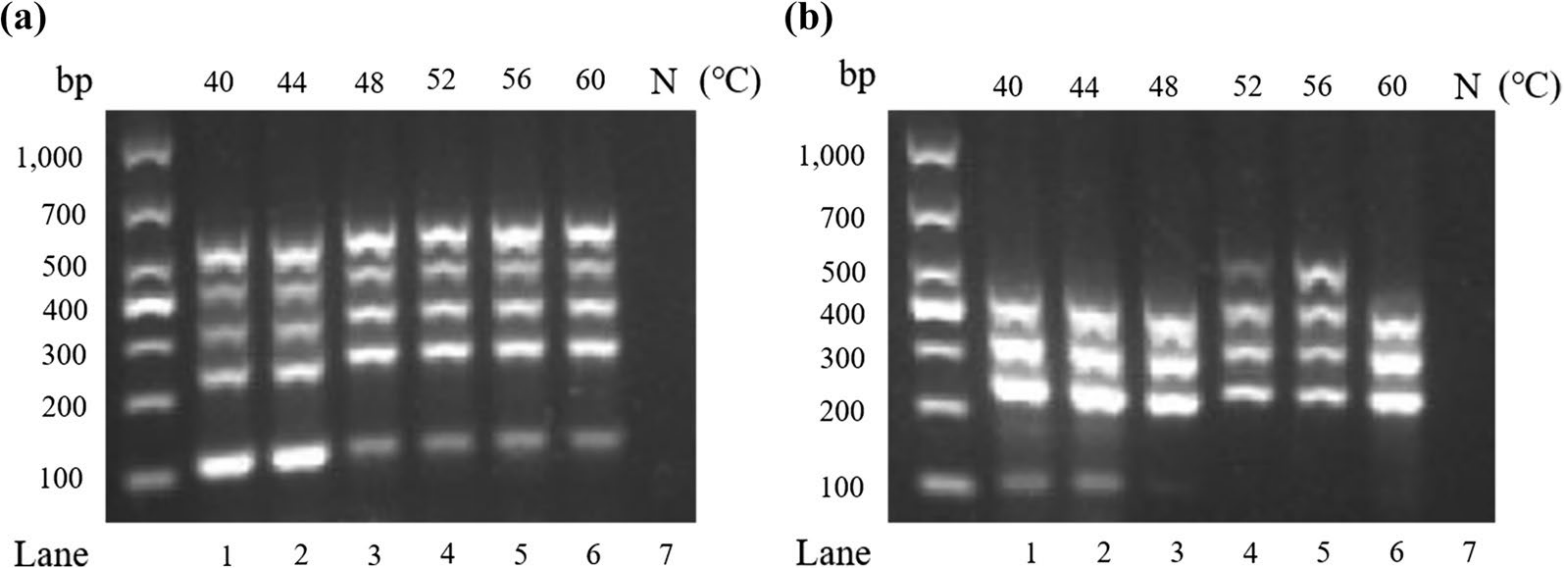
Annealing temperature optimization comparison between DPO primers and conventional primers. Lanes 1–6 represent 40 °C, 44 °C, 48 °C, 52 °C, 56 °C, 60 °C, for a gradient with 6 temperatures; Lane 7 represents negative template (N). **a** DPO primer annealing temperature optimization test; **b** Normal annealing temperature optimization test for primers

In a comparison of DPO primers with conventional primers, it was found that DPO primers have a wide range of effective annealing temperatures (Fig. 3a), with no significant differences in bands, while conventional primers have the best annealing temperature (Fig. 3b). These results show that DPO primers were not sensitive to annealing temperature.

### Specificity of the DPO system-based multiplex RT-PCR

**Fig. 4:**
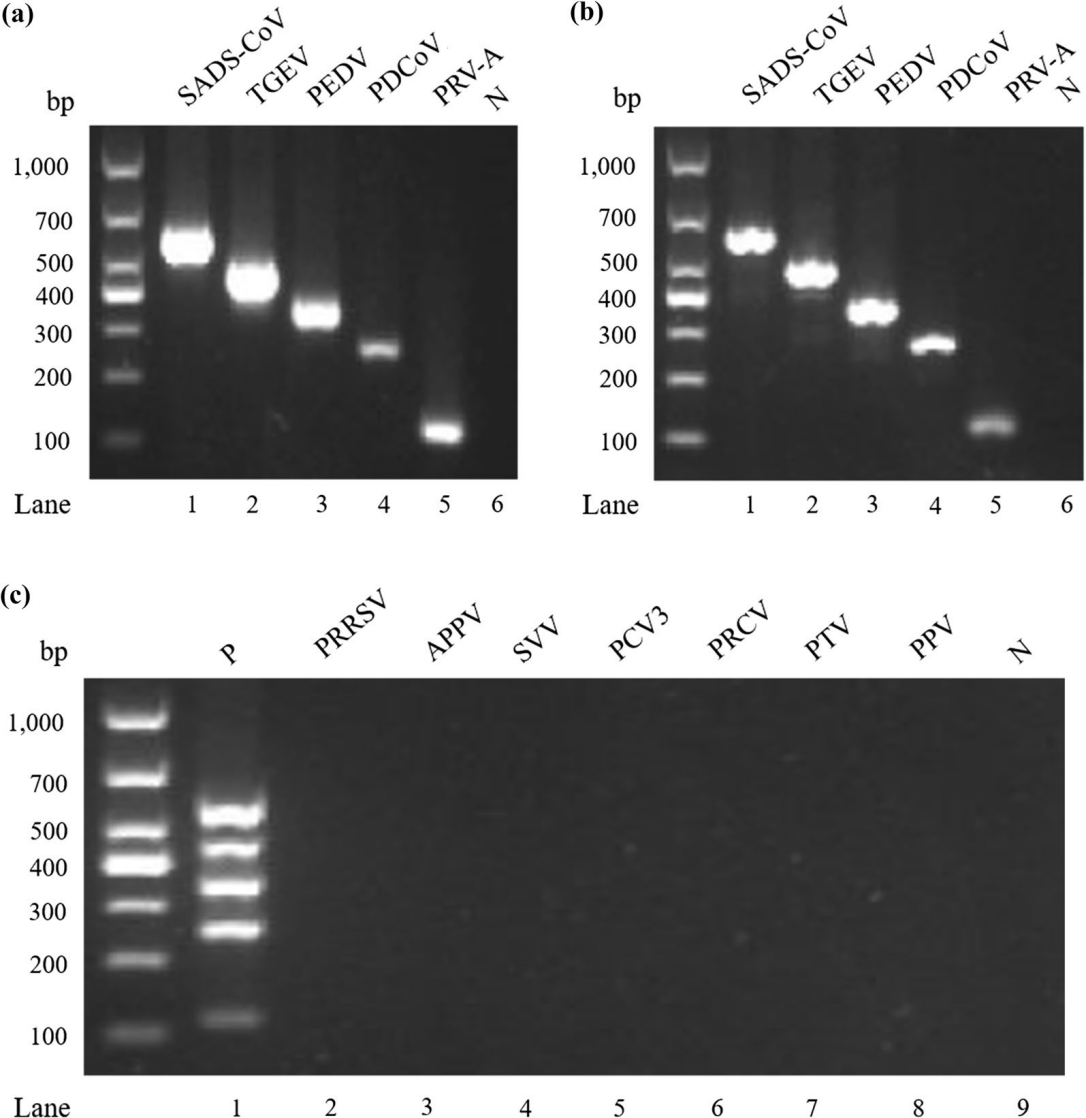
Detection specificity of SADS-CoV, TGEV, PEDV, PDCoV, and PRV-A by the DPO system-based multiplex RT-PCR. **a** A single pair of the DPO primer was used to amplify the mixture of the five plasmid templates. (Lanes 1–6: SADS-CoV, TGEV, PEDV, PDCoV, PRV-A, and negative control). **b** The mixture of the five DPO primers was used with each of the five plasmid templates individually, for amplification (Lanes 1–6: SADS-CoV, TGEV, PEDV, PDCoV, PRV-A, and negative control). **c** Detection of the other diarrhea viruses (Lanes 1–9: positive control (P), PRRSV, APPV, SVV, PCV3, PRCV, PTV, PPV, and negative template)

The specificity of the DPO system-based multiplex RT-PCR was determined by amplifying the mixture of the SADS-CoV, TGEV, PEDV, PDCoV, and PRV-A template with a single pair of DPO primers. Each pair of DPO primers produced bands corresponding to the expected size (Fig. 4a), and no other non-specific bands were present. A mixture of the five pairs of DPO primers was used to amplify each virus template. The results of the agarose gel electrophoresis showed DPO system-based multiplex RT-PCR amplification for each band containing SADS-CoV, TGEV, PEDV, PDCoV, and PRV-A (Fig. 4b). With the DPO system-based multiplex primers, other common swine viruses—such as PRRSV, APPV, SVV, PCV3, PRCV, PTV, and PPV virus cDNA—did not produce any bands (Fig. 4c).

### Sensitivity of the DPO system-based multiplex RT-PCR

**Fig. 5:**
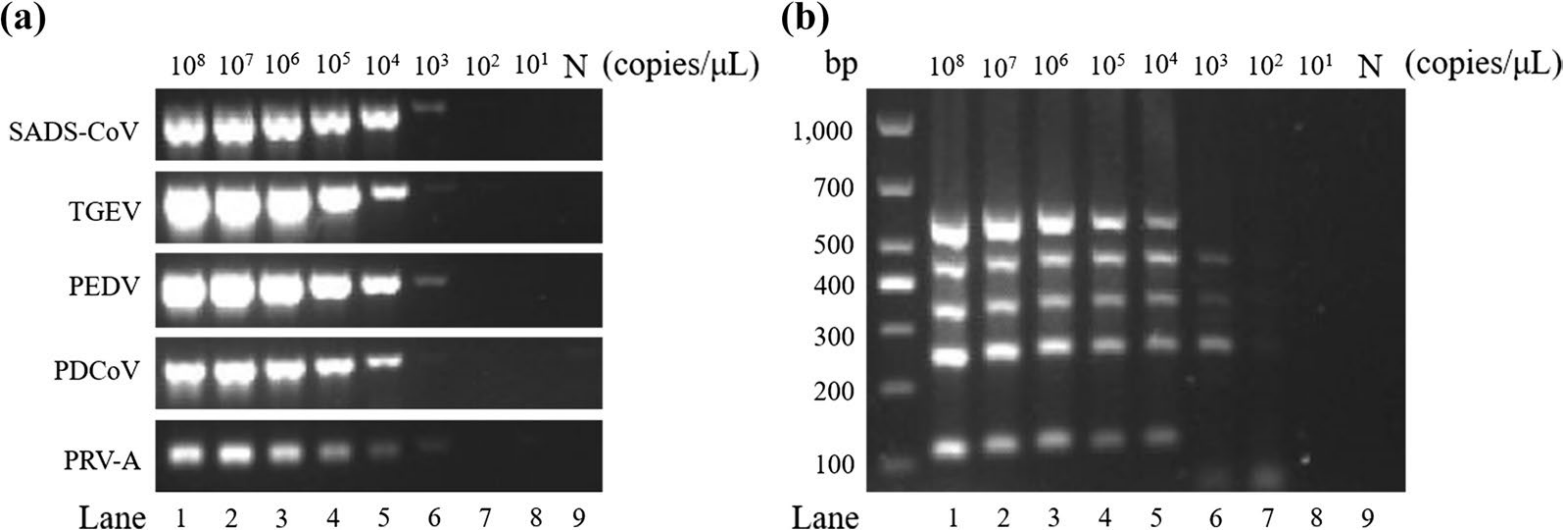
Detection sensitivity of SADS-CoV, TGEV, PEDV, PDCoV, and PRV-A by the DPO system-based multiplex RT-PCR. **a** Standard plasmids of TGEV, PEDV, PDCoV, SADS-CoV, and PRV-A were diluted tenfold to a final concentration between 10^8^ copies/μL and 10^1^ copies/μL. The cDNAs were amplified with specific DPO primer sets. **b** The standard plasmids of each virus were incorporated after dilution and then added to a tube containing the DPO primer mixture for PCR amplification

In order to evaluate the sensitivity of the DPO system-based multiplex RT-PCR detection method, the sensitivity of a single RT-PCR of the DPO system-based assay for each virus was established. For the single RT-PCR, the limits of SADS-CoV, TGEV, PEDV, PDCoV, and PRV-A were 6.12 × 10^4^ copies/μL, 8.88 × 10^3^ copies/μL, 6.84 × 10^3^ copies/μL, 7.47 × 10^3^ copies/μL, and 6.51 × 10^3^ copies/μL (Fig. 5a), respectively. With the multiplex RT-PCR based on the DPO system, the sensitivities of SADS-CoV, TGEV, PEDV, PDCoV, and PRV-A were 6.12 × 10^4^ copies/μL, 8.88 × 10^3^ copies/μL, 6.84 × 10^3^ copies/μL, 7.47 × 10^3^ copies/μL, and 6.51 × 10^4^ copies/μL (Fig. 5b), respectively.

### Reproducibility of the DPO system-based multiplex RT-PCR

To evaluate the reproducibility of the DPO system-based multiplex RT-PCR, three repeat experiments were conducted with an interval of 45 days (Additional file 1: Figure S1). The experimental results showed that the three samples of the different batches have good repeatability; each batch of samples displayed consistent target bands, which shows that the method is repeatable.

### Detection in the clinical samples

To evaluate the DPO system-based multiplex RT-PCR for detecting virus infection in swine, 181 swine intestinal samples collected from four provinces in South China were assayed as well as confirmed by an RT-PCR method. The results showed that SADS-CoV (11.6%, 21/181), PEDV (30.94%, 56/181), PDCoV (17.67%, 32/181), PRV-A (9.39%, 17/181), and TGEV (0.55%, 1/181) existed in pig herds, and these were consistent with the single RT-PCR (Table 2). Coinfection of two or more viruses was also common, with such combinations as SADS-CoV + PEDV, SADS-CoV + PDCoV, PEDV + PDCoV, PEDV + PRV-A, PDCoV + PRV-A, SADS-CoV + PEDV + PDCoV, SADS-CoV + PEDV + PRV-A, and SADS-CoV + PEDV + PDCoV + PRV-A. PEDV had the highest positive rate at 30.94% (56/181). TGEV had the lowest positive rate at 0.55% (1/181). The cases of co-infection of PEDV and PDCoV were more with the rate of 9.39% (17/181) (Table 3).

**Table 2:** Clinical application of the conventional RT-PCR and DPO system-based multiplex RT-PCR detection method

Detection method	SADS-CoV	TGEV	PEDV	PDCoV	PRV-A	Coincidence rate of detection method/%
RT-PCR	11.6% (21/181)	0.55% (1/181)	30.94% (56/181)	17.67% (32/181)	9.39% (17/181)	100%
DPO RT-PCR	11.6% (21/181)	0.55% (1/181)	30.94% (56/181)	17.67% (32/181)	9.39% (17/181)

**Table 3:** Results of clinical samples detected by the multiplex RT-PCR based on the DPO system

Year	One pathogen positive samples					Two pathogens positive samples					Three pathogens positive samples		Four pathogens positive samples
	SADS-CoV	PEDV	PDCoV	PRV-A	TGEV	SADS-CoV + PEDV	SADS-CoV + PDCoV	PEDV + PDCoV	PEDV + PRV-A	PDCoV + PRV-A	SADS-CoV + PEDV+ PDCoV	SADS-CoV + PEDV + PRV-A	SADS-CoV + PEDV + PDCoV + PRV-A
2016	2	10	5	4	0	5	1	7	3	1	2	0	1
2017	3	9	5	5	1	2	0	1	0	1	1	1	1
2018	1	9	2	0	0	0	0	4	0	0	0	0	0
Total	7 (3.87%)	28 (15.47%)	12 (6.63%)	9 (4.97%)	1 (0.55%)	7 (3.87%)	1 (0.55%)	12 (6.63%)	3 (1.66%)	2 (1.1%)	3 (1.66%)	1 (0.55%)	2 (1.1%)

## Discussion

Diarrheal virus infection causes diarrhea, high mortality in piglets, and tremendous economic losses for swine farms worldwide. All swine enteric viruses—including TGEV, PEDV, PRV-A, and new variant strains PDCoV and SADS-CoV(Bevins et al. 2018; Gong et al. 2017; Pan et al. 2017; Zhang et al. 2015; Zhao et al. 2019)—could seriously endanger the development of the pig industry, especially in terms of newborn piglets. The symptoms caused by the above mentioned five viruses are similar, so it is difficult to determine the causative pathogen in clinical diagnosis. Moreover, relatively few studies examine newly-epidemic diseases. Therefore, a rapid, specific, and low-cost detection method is sorely needed for the surveillance of diarrhea viruses.

In recent years, the methods of conventional RT-PCR, multiplex RT-PCR, and multiplex RT-qPCR targeting conservative regions were developed (Ding et al. 2020; Huang et al. 2019). The method based on RT-qPCR has certain shortcomings, such as high cost and high requirements for instruments, and many laboratories are unable to acquire the relatively expensive qPCR machine. Multiplex RT-PCR can simultaneously detect multiple pathogenic nucleic acids in one reaction tube, which improves detection efficiency; however, in traditional multiple RT-PCR systems, the formation of dimers between multiple primers and the mismatch of primers and templates affect the sensitivity and the specificity of the detection method (Ma et al. 2015).

As a new primer design method, DPO primers have a special structure that makes it difficult to form a secondary structure, so primers do not need to be screened in the experiment. Additionally, the structure is insensitive to the annealing temperature and does not require optimization of the annealing temperature, which simplifies the primer design and the experimental steps, improves the detection efficiency, and provides new prospects for application of multiple PCR technology. To our knowledge, no previous method based on the DPO system has been developed to detect and distinguish TGEV, PEDV, PRV-A, PDCoV, and SADS-CoV simultaneously in one tube. Thus, we developed the DPO system-based multiplex RT-PCR for detection and differential diagnosis of five diarrhea viruses in pig herds.

The DPO system-based multiplex RT-PCR detection method established in this study can achieve efficient amplification of target genes in the annealing temperature range of 40–60 °C. Optimization of the annealing temperature is not necessary, compared to conventional PCR primers. The result of the experiment can be obtained in a mere three hours. The specificity of the test was evaluated in three ways. The results demonstrated that each DPO primer can only detect the target gene itself and cannot detect non-specific fragments; this is consistent with a previous study, which showed that the DPO system prevents non-specific amplification without inhibiting the effective amplification of the target band (Chun et al. 2007). The sensitivity results showed that the limit of two target genes in the DPO system-based multiplex RT-PCR was lower than in the single RT-PCR. Competition may occur among primers, templates, and reagents. In detection in clinical samples, the coincidence rate of the two methods was 100%.

According to this study’s test results, PEDV (30.94%, 56/181) was still the primary cause of swine diarrhea in South China, which was consistent with previous studies (Sun et al. 2012; Zhou et al. 2018). Swine viral diarrhea may be caused by one virus but also by a mixture of several viruses. Zhou et al. reported that coinfections of swine enteric viruses were common in pig farms in China (Zhou et al. 2018). We confirmed mixed infections of two or more viruses in clinical practice. Among the mixed infections, the coinfection rate of PEDV and PDCoV (9.39%, 17/181) was the highest, followed by PEDV and SADS-CoV (7.18%, 13/181). The current investigation showed that SADS-CoV was only detected in Guangdong, but not in other areas; previous studies found it to exist in Fujian (Li et al. 2018), so it is necessary to strengthen the monitoring of SADS-CoV to prevent it from spreading to other areas. Previous studies have shown that the coronavirus has extensive in vitro host tropism and can infect a variety of cell lines (Edwards et al. 2020; Luo et al. 2020; Yang et al. 2020, 2019). At the same time, mixed infections may lead to recombinations between viruses and changes in the virulence of the virus (Akimkin et al. 2016; Ding et al. 2020). This highlights the importance of identifying multiple co-occurring virus infections.

In summary, we developed a DPO system-based multiplex RT-PCR with high specificity and high detection efficiency for application in epidemiological studies, laboratory diagnosis, and surveillance of SADS-CoV, TGEV, PEDV, PDCoV, and PRV-A. Moreover, the established method can be applied to the clinical differential diagnosis of mixed infections for early diagnosis in clinical cases.

## Supplementary Information

**Additional file 1: Figure S1.** Repeatability experiment of the DPO system-based multiplex RT-PCR. Three repeated experiments were carried out, with an interval of 45 days. (a): The first repetition; (b): The second repetition; (c): The third repetition.

## Data Availability

We will make data fully available and without restriction.

## References

[CR1] Akimkin V, Beer M, Blome S, Hanke D, Höper D, Jenckel M, Pohlmann A (2016). New chimeric porcine coronavirus in Swine Feces, Germany, 2012. Emerg Infect Dis.

[CR2] Bevins SN, Lutman M, Pedersen K, Barrett N, Gidlewski T, Deliberto TJ, Franklin AB (2018). Spillover of Swine coronaviruses, United States. Emerg Infect Dis.

[CR3] Chun JY, Kim KJ, Hwang IT, Kim YJ, Lee DH, Lee IK, Kim JK (2007). Dual priming oligonucleotide system for the multiplex detection of respiratory viruses and SNP genotyping of CYP2C19 gene. Nucleic Acids Res.

[CR4] Ding G, Fu Y, Li B, Chen J, Wang J, Yin B, Sha W, Liu G (2020). Development of a multiplex RT-PCR for the detection of major diarrhoeal viruses in pig herds in China. Transbound Emerg Dis.

[CR5] Edwards CE, Yount BL, Graham RL, Leist SR, Hou YJ, Dinnon KH, Sims AC, Swanstrom J, Gully K, Scobey TD, Cooley MR, Currie CG, Randell SH, Baric RS (2020). Swine acute diarrhea syndrome coronavirus replication in primary human cells reveals potential susceptibility to infection. Proc Natl Acad Sci U S A.

[CR6] Fan WL, Wang ZW, Qin Y, Sun C, Liu ZM, Jiang YP, Qiao XY, Tang LJ, Li YJ, Xu YG (2017). Use of dual priming oligonucleotide system-based multiplex RT-PCR combined with high performance liquid chromatography assay for simultaneous detection of five enteric viruses associated with acute enteritis. J Virol Methods.

[CR7] Gong L, Li J, Zhou Q, Xu Z, Chen L, Zhang Y, Xue C, Wen Z, Cao Y (2017). A New Bat-HKU2-like Coronavirus in Swine, China, 2017. Emerg Infect Dis.

[CR8] Huang X, Chen J, Yao G, Guo Q, Wang J, Liu G (2019). A TaqMan-probe-based multiplex real-time RT-qPCR for simultaneous detection of porcine enteric coronaviruses. Appl Microbiol Biotechnol.

[CR9] Kim JK, Lee HJ, Lee YJ, Chun JY, Lee IK, Lim YS, Suh DJ, Ko SY, Kim MH, Oh HB (2008). Direct detection of lamivudine-resistant hepatitis B virus mutants by a multiplex PCR using dual-priming oligonucleotide primers. J Virol Methods.

[CR10] Li W, Li H, Liu Y, Pan Y, Deng F, Song Y, Tang X, He Q (2012). New variants of porcine epidemic diarrhea virus, China, 2011. Emerg Infect Dis.

[CR11] Li G, Chen Q, Harmon KM, Yoon KJ, Schwartz KJ, Hoogland MJ, Gauger PC, Main RG, Zhang J (2014). Full-length genome sequence of porcine deltacoronavirus strain USA/IA/2014/8734. Genome Announc.

[CR12] Li K, Li H, Bi Z, Gu J, Gong W, Luo S, Zhang F, Song D, Ye Y, Tang Y (2018). Complete genome sequence of a novel swine acute diarrhea syndrome coronavirus, CH/FJWT/2018, isolated in Fujian, China, in 2018. Microbiol Resour Announc.

[CR13] Luo Y, Chen Y, Geng R, Li B, Chen J, Zhao K, Zheng XS, Zhang W, Zhou P, Yang XL, Shi ZL (2020). Broad cell tropism of SADS-CoV in vitro implies its potential cross-species infection risk. Virol Sin.

[CR14] Ma X, Xu H, Shi L, Yang P, Zhang L, Sun X, Zhen W, Hu K (2015). A multiplex PCR assay for the detection of five influenza viruses using a dual priming oligonucleotide system. BMC Infect Dis.

[CR15] Pan Y, Tian X, Qin P, Wang B, Zhao P, Yang YL, Wang L, Wang D, Song Y, Zhang X, Huang YW (2017). Discovery of a novel swine enteric alphacoronavirus (SeACoV) in southern China. Vet Microbiol.

[CR16] Sun RQ, Cai RJ, Chen YQ, Liang PS, Chen DK, Song CX (2012). Outbreak of porcine epidemic diarrhea in suckling piglets. China Emerg Infect Dis.

[CR17] Woo PC, Lau SK, Lam CS, Lau CC, Tsang AK, Lau JH, Bai R, Teng JL, Tsang CC, Wang M, Zheng BJ, Chan KH, Yuen KY (2012). Discovery of seven novel Mammalian and avian coronaviruses in the genus deltacoronavirus supports bat coronaviruses as the gene source of alphacoronavirus and betacoronavirus and avian coronaviruses as the gene source of gammacoronavirus and deltacoronavirus. J Virol.

[CR18] Xu Z, Zhang Y, Gong L, Huang L, Lin Y, Qin J, Du Y, Zhou Q, Xue C, Cao Y (2019). Isolation and characterization of a highly pathogenic strain of Porcine enteric alphacoronavirus causing watery diarrhoea and high mortality in newborn piglets. Transbound Emerg Dis.

[CR19] Yang YL, Qin P, Wang B, Liu Y, Xu GH, Peng L, Zhou J, Zhu SJ, Huang YW (2019). Broad cross-species infection of cultured cells by bat HKU2-related swine acute diarrhea syndrome coronavirus and identification of its replication in murine dendritic cells in vivo highlight its potential for diverse interspecies transmission. J Virol.

[CR20] Yang Y-L, Yu J-Q, Huang Y-W (2020). Swine enteric alphacoronavirus (swine acute diarrhea syndrome coronavirus): an update three years after its discovery. Virus Res.

[CR21] Zhang H, Zhang Z, Wang Y, Wang X, Xia M, Wu H (2015). Isolation, molecular characterization and evaluation of the pathogenicity of a porcine rotavirus isolated from Jiangsu Province. China Arch Virol.

[CR22] Zhang J, Tsai YL, Lee PY, Chen Q, Zhang Y, Chiang CJ, Shen YH, Li FC, Chang HF, Gauger PC, Harmon KM, Wang HT (2016). Evaluation of two singleplex reverse transcription-Insulated isothermal PCR tests and a duplex real-time RT-PCR test for the detection of porcine epidemic diarrhea virus and porcine deltacoronavirus. J Virol Methods.

[CR23] Zhang H, Liang Q, Li B, Cui X, Wei X, Ding Q, Wang Y, Hu H (2019). Prevalence, phylogenetic and evolutionary analysis of porcine deltacoronavirus in Henan province, China. Prev Vet Med.

[CR24] Zhao Y, Qu H, Hu J, Fu J, Chen R, Li C, Cao S, Wen Y, Wu R, Zhao Q, Yan Q, Wen X, Huang X (2019). Characterization and pathogenicity of the porcine deltacoronavirus isolated in Southwest China. Viruses.

[CR25] Zhou P, Fan H, Lan T, Yang XL, Shi WF, Zhang W, Zhu Y, Zhang YW, Xie QM, Mani S, Zheng XS, Li B, Li JM, Guo H, Pei GQ, An XP, Chen JW, Zhou L, Mai KJ, Wu ZX, Li D, Anderson DE, Zhang LB, Li SY, Mi ZQ, He TT, Cong F, Guo PJ, Huang R, Luo Y, Liu XL, Chen J, Huang Y, Sun Q, Zhang XL, Wang YY, Xing SZ, Chen YS, Sun Y, Li J, Daszak P, Wang LF, Shi ZL, Tong YG, Ma JY (2018). Fatal swine acute diarrhoea syndrome caused by an HKU2-related coronavirus of bat origin. Nature.

[CR26] Zhou L, Sun Y, Lan T, Wu R, Chen J, Wu Z, Xie Q, Zhang X, Ma J (2019). Retrospective detection and phylogenetic analysis of swine acute diarrhoea syndrome coronavirus in pigs in southern China. Transbound Emerg Dis.

